# Phishing simulation exercise in a large hospital: A case study

**DOI:** 10.1177/20552076221081716

**Published:** 2022-03-16

**Authors:** Fabio Rizzoni, Sabina Magalini, Alessandra Casaroli, Pasquale Mari, Matt Dixon, Lynne Coventry

**Affiliations:** 1 Data Protection Office, Fondazione Policlinico Gemelli, Italy; 2 Department of Surgery, 9371 Catholic University of the Sacred Heart, Italy; 3 Information Communication Technology Service, 18654 Fondazione Policlinico Gemelli, Italy; 4 Department of Psychology, 5995 Northumbria University, UK

**Keywords:** Cybersecurity, training, technology

## Abstract

**Background:**

Phishing is a major threat to the data and infrastructure of healthcare organizations and many cyberattacks utilize this socially engineered pathway. Phishing simulation is used to identify weaknesses and risks in the human defences of organizations. There are many factors influencing the difficulty of detecting a phishing email including fatigue and the nature of the deceptive message.

**Method:**

A major Italian Hospital with over 6000 healthcare staff performed a phishing simulation as part of its annual training and risk assessment. Three campaigns were launched at approx. 4-month intervals, to compare staff reaction to a general phishing email and a customized one.

**Results:**

The results show that customization of phishing emails makes them much more likely to be acted on. In the first campaign, 64% of staff did not open the general phish, significantly more than the 38% that did not open the custom phish. A significant difference was also found for the click rate, with significantly more staff clicking on the custom phish. However, the campaigns could not be run as intended, due to issues raised within the organization.

**Conclusions:**

Phishing simulation is useful but not without its limitations. It requires contextual knowledge, skill and experience to ensure that it is effective. The exercise raised many issues within the Hospital. Successful, ethical phishing simulations require coordination across the organization, precise timing and lack of staff awareness. This can be complex to coordinate. Misleading messages containing false threats or promises can cause a backlash from staff and unions. The effectiveness of the message is dependent on the personalization of the message to current, local events. The lessons learned can be useful for other hospitals.

## Introduction

The COVID-19 pandemic saw an increase in phishing attacks in general^1^ and targeted at the healthcare sector specifically.^2^ Phishing is a form of deception in which the attacker sends a fraudulent message designed to trick a human target into revealing sensitive information or to enable malicious software such as ransomware to infiltrate the target's infrastructure. Phishing is typically carried out through email,^3,4^ though less common forms exist through SMS (Smishing) or Voice calls (Vishing). Phishing has become a global everyday threat for Healthcare organizations in the last few years.^5,6^ The COVID pandemic has further exacerbated the situation, with varying reports estimating a range from a 600% to 9000% increase in phishing attacks.^7^ Phishing is versatile, requiring little technical knowledge and most of all use vulnerabilities that are very difficult to patch – those of human behaviour. While email filtering systems claim to accurately detect a very high proportion of phishing emails,^8,9^ a small number will inevitably always reach users, leaving human decision making as a further point of vulnerability. Phishing has become the most common way to spread malware and launch attacks which can devastate organizations and lead to the compromise of highly sensitive data.

Spear phishing is also on the increase, attacking individuals via a personalized phishing email. Despite this, there is a noticeable lack of real-work studies of phishing in organizations, where the attack is customized for a particular organization. Among healthcare organizations, hospitals are particularly vulnerable to phishing attacks as it is difficult for management to enforce a strict cybersecurity policy^10^ and staff may miss the signs of a phishing email as a result of fatigue, being more focused on patient care than administration tasks,^11^ or simply because phishing emails are hard to detect. If they followed a recognizable pattern – security developers would be able to write software to filter them out and would not need to rely on human intervention. While some headway in automatic detection has been made by placing suspicious emails in junk mail, too often legitimate emails are also captured in this filter^12^ while some continue to evade detection. This reduces trust in the reliability of junk mail filtering.

A phishing simulation is an authorized simulated attack that evaluates staff's ability to recognize phishing email attacks. Phishing simulations are available from many cyber awareness training companies and have been in the subject of several research studies^5,13–15^ which aim to develop an understanding of how certain characteristics of phishing emails (e.g. use of authority and urgency) can influence the susceptibility of users to these emails. A large proportion of phishing simulations are deployed in occupational settings, as a means of identifying an organization's overall phishing vulnerability, and/or as a test of the efficacy of security training provided in the workplace.^13,14,16^ However, there is little research into how to best design a phishing simulation to enable employees to effectively build resilience against such attacks, subsequently, such simulations have met with mixed success. For example, Gordon et al.^13^ showed that mandatory training is not always successful in reducing the number of staff who click on new phishing emails in healthcare. However, he did find that repeated exposure to phishing campaigns can reduce the likelihood of clicking on a specific, known attack, as it becomes familiar and recognizable.

Despite their mixed success, phishing simulations are generally considered an efficient method of testing the phishing awareness of large user groups. Firstly, they allow a broad workforce to be tested simultaneously without scheduling time away from work to take part. Further, phishing simulations ensure that the skills and awareness of the users are tested in a naturalistic setting; users should hypothetically act in the same way as if they received a real phishing email, offering a realistic representation of the anti-phish behaviour of users/staff. However, such methods can be ethically questionable. Those receiving a simulated phishing email are not typically made aware of the test in advance. This is required to ensure that staff are not undertaking higher levels of surveillance and alertfulness than normal. In research contexts, this causes an issue around obtaining informed consent; participants are not made fully aware of the activities they are participating in,^17^ leaving many participants feeling angry and deceived, having unwittingly taken part in a simulated phishing attack.^18^

While consent is not required for a workplace, in so far as these simulations are positioned as training and/or a risk assessment, similar feeling of anger and mistrust at the clandestine nature of the testing and staff may feel they are being unduly surveilled. This can damage the morale and interpersonal trust, and trust in the organization, depending on the context of the simulation,^19,20^ which can reduce productivity.^21^ Other negative consequences, such as employees disengaging from official, future correspondence due to fear of being caught out by a phishing test^22^ and fear of being sacked if they fail the test^23^ are also possible. As such, while phishing simulation exercises may be an effective way to understand the phishing awareness of a workforce, they can be harmful to the workplace environment and must be managed carefully.

## Related work

Hospitals and other healthcare settings are a priority setting in which to understand cybersecurity vulnerabilities, given the high-risk nature of the sensitive data handled through these organizations, as well as the critical services they deliver. A narrative review of this area concludes that the key areas that must be developed are Human Behaviour, the available Technology and Internal Processes of organizations around cybersecurity. The current study focuses on the human behaviour aspect, namely, how employees may fall victim to phishing emails. Below, research investigating the human behaviour and attitudes around cybersecurity are discussed.

Jalali et al.^6^ investigated employee phishing threat perceptions in a healthcare setting, along with the Theory of Planned Behaviour. Their findings suggest that the intentions of staff to follow security protocols do not significantly influence an employee's rate of clicking on simulated phishing emails – however, there was a significant positive correlation between employee workload and phishing vulnerability. In other words, staff fully intended to detect phishing attacks, but were not able to do so, and the higher their workload and fatigue, the less likely they were to detect such attacks. This finding is corroborated by qualitative work which suggests hospital staff simply cannot prioritize rigorous cybersecurity behaviours over their duties to provide care for participants.^24^ Indeed, hospital staff are widely known to be overworked, with a high prevalence of burnout experienced.^25,26^ Given research has shown that cybersecurity is not prioritized over healthcare demands,^24^ we can conclude that increasing demands on healthcare staff, especially due to crises such as the COVID-19 pandemic may lead to increased cybersecurity vulnerability and incidents. Work which aims to pragmatically address these vulnerabilities is critical. Below we discuss research that has employed phishing simulations and training exercises as a means to understand the specific phishing vulnerability of hospitals.

Some research simply aims to quantify the extent to which phishing emails are engaged with in simulations. Some studies show rates of about one in seven phishing emails being clicked by employees, however, more concerning findings show that approximately 16% of staff go as far as downloading an attachment from a simulated phishing email and only 32% of mandated reporters actually report suspicious emails to IT staff.^27^ Further work which builds on this tests the efficacy of training programs aimed at those who fail phishing simulation tasks, though results suggest such programs are not especially successful,^13^ in spite of previous work which has found anti-phishing training to improve user performance against phishing emails.^14^ As Gordon et al.^5,13^ find across two studies, the only decrease in employee click rates was due to time and repeated exposure o phishing simulation exercises. These findings suggest that hospital staff do appear significantly vulnerable to phishing attacks, yet typical training methods are not sufficient to address this – thus the healthcare setting appears to be unique in its vulnerabilities and requires further in-depth study. One potential means to reduce phishing vulnerability may be to repeatedly expose users to phishing simulations. However, further work is needed to develop the phishing simulation process – the current study aims to build on previous findings on phishing simulations to consider how best we can develop phishing simulations for a healthcare context, while avoiding the issues discussed above which may add further stress to individuals in high-stress roles, or even breed ambivalence between medical and IT staff.

This paper presents a real-world case study of a yearlong, phishing simulation carried out in a major Italian hospital with over 6000 employees and documents both positive and negative aspects of this experience. A context-specific phishing email was compared to a general phishing email from a phishing simulation provider. Data on the phishing attack susceptibility, as measured by click rates, were collected at three points in the year. Indicative statistics and a narrative summary of the issues relating to managing this campaign are provided to communicate the implementation context and the encountered criticalities starting from the reasons which led to the initiative and ending with the results achieved.

## The context

This paper reports on a phishing campaign organized by a consultancy in an Italian hospital. The hospital is a major Italian Hospital with over 1500 beds and over 6000 employees. Infrastructural capacities are in place and align with all Italian requirements. Information Technology systems comply with all required standards. The hospital is currently involved with a European Project, PANACEA (www.panacearesearch.eu), developing a holistic approach to cybersecurity that studies both the technical and human factors influencing the hospital's defences against cybercrime. This work identified fear of phishing attacks from staff, but a lack of confidence in their ability to recognize such an attack.^24^ The phishing simulation was implemented in response to this knowledge and an attempt to defraud the organization via a whaling attack, where the attacker pretended to be the Director of the Hospital and the target was the Head of Purchasing. Luckily the target was suspicious of the request to transfer money and sought to contact the Director via another communication channel.

Online mandatory training for cybersecurity is already implemented on the Hospital's Intranet site, and technological mitigation is in place and running but out of the scope of this paper. Initially, a graphical phishing awareness message was placed on the Hospital's intranet homepage indicating the risks of phishing and highlighting the correct behaviour. But these approaches are limited by the very nature of didactic tasks which are perceived as onerous and create conscious and unconscious psychological resistance.^28^ It is well known that medical staff in healthcare settings do not have time to follow extracurricular activities and courses on security training within the timelines dictated by the Management.^10^ In fact, the data available from the hospital's intranet site showed that over a third of the medical staff had not performed the recent Privacy and GDRP e-training requested by the Data Protection Officer within the expected timeframe. Therefore, a phishing simulation was introduced to provide an efficient and rapid assessment of the level of risk to the hospital posed by phishing and to bring the issue to the general attention of staff and hopefully nudge staff to be more vigilant.

## Participants

The majority of staff with a hospital email account received phishing emails. In each campaign, before the start of the campaigns, an email list was created of all current included staff. This was used throughout the campaigns. While some staff may have left during the time period, no new staff were added to the email list. Individual staff were not tracked over the three campaigns. Staff were randomly assigned to either the standard or the customized phishing email in the first campaign. In the first campaign, 5313 staff received an email, in the second 2700, and in the third 5198. Details of the split can be found in the procedure. Staff included administrative, nursing, and medical personnel. Top management, both administrative and academic, were excluded as they were aware of the campaign.

## Materials

All phishing emails were sent from the same fake sender, with slight changes on the hospital’s email address. The Hospital is a first-level national domain (‘.it’), the phishing e-mails were sent from ‘.com’. The following phishing emails were used:

Campaign 1, general phish: March 2019 an email saying that a Microsoft email about pay scales had been put in quarantine and to click on the link and provide their password to get it out of quarantine. The text was an image rather than text and presented using very poor grammar.

Campaign 1, customized phish: March 2019 an email saying that they had 48 h to complete mandatory online training and to click on the link to begin the training. This was based on current activities in the hospital and contained some grammatical errors.
Campaign 2, customized phish: on 11 December 2019 an email said that a Christmas bonus would be paid on the 18th December but it must be claimed by clicking on the link in the email. There were no apparent grammatical errors.
Campaign 3, combined phish: September 2020 an email offering free dropbox upgrade in thanks for their support during COVID-19, if a link clicked before December. In this case, the text was an image rather than text.

## Procedure

The original simulation project planned to dispatch simulated phishing emails, through an external consultancy, to all the Hospital's personnel. The project was to last 1 year from 2019 to 2020 and the dispatch of the mails was to take place over three campaigns. The consultancy company, considering previous experience with other clients, proposed a 4-month interval between the campaigns. The consultancy generally used a proprietary, generic phishing email (standard email). However, the hospital's internal working group decided to add a more tailored version (custom email) to more specifically target hospital staff. Two messages (one standard and one custom) were to be sent out in each campaign. Each of the six emails contained a different link associated with the same HTML landing page. Whoever clicked on the link would be forwarded to a page containing an explanation of the phishing exercise and a list of eight ways to avoid this happening again in the future.

First staff were reminded that phishing emails are difficult to detect and that the technology cannot currently detect them, and so their observation and reaction are vital. The eight points informed staff of the correct domain for the hospital, not to click on any email they are worried about, never to insert a PIN or password if directed to a page from an email, how to check the underlying URL by hovering the mouse, not to open files from a suspicious email, not to use work email for non-work purposes, be aware of emails that are marked as urgent and be aware of how social media can be used to personalize emails.

Tracking of who opened the email was carried out by the consultancy's backend software. While this is a consistent measure, it may systematically under-report the number of emails that are opened. However, the number of receivers who click the link is accurate. Data was managed via the external consultancy to protect the identification of individuals within the organization that had fallen for the Phish. The hospital was only provided with summary data.

This original project encountered technical and logistic difficulties and had to be modified across campaigns. This resulted in certain parts of the campaign not being activated. The final setup was as follows:

First campaign (August 2019): 2656 staff received standard phishing emails and 2657 received a customized version.
Second campaign (December 2019): Customised phishing email sent to 2700 staff. No message was sent to the other group.
Third campaign (April 2020): 5198 staff received a modified standard mail.

The campaigns proceeded as follows:

### First campaign

In the first campaign, half of the staff received a standard email, and half a customized email. 5400 email addresses were randomly assigned to one of the two email groups. The standard group received a noticeably unprofessional email translated by an automatic translator that requested the target save a phantom email that was in quarantine through an incongruous insertion of the user's password. The sender was a non-existing risorse.dipendenti@censura.com. This email was evidently incongruous and its purpose was essentially to measure the users’ preparedness level, especially for the second campaign.

The customized email group received an email that was customized to the present hospital activities. As mentioned earlier, any members of staff still had to complete an online security training course. The customized email utilized this context and requested the receiver to click on, what looked like, the online course link. The email could be identified as false because of the unusual sender risorse.dipendenti@censura.com, lack of punctuation and a very evident grammatical error. The sender and grammatical issues were consistent across the two emails.

Table 1 provides details of the mail sent and the overall response to these emails during the first campaign.

**Table 1.** Details of messages sent and responses in the first campaign.

First campaign	Standard email		Custom email	
Total emails sent	2656	100%	2657	100%
Received not opened	1699	64%	1012	38%
Received and opened	957	36%	1645	62%
Received, opened and link clicked	176	7% of emails sent 18% of opened,	1447	55% of emails sent 88% of opened

### Second campaign

This campaign met with some problems. Interestingly, prior to this campaign, the upgrade to the anti-virus system meant that 1600 emails went straight into the junk-mail, thus detection of spam had clearly improved and added an extra indicator to staff that this email was potentially problematic.

The second set of emails was sent 5 months after the first set, during the Christmas season. The second set was designed in the same way as the first but with three differences. Firstly, those that had received the standard email received the custom email and vice versa. This meant that 18% of those receiving the custom phishing mail had been told that they had clicked on a phishing link and received the information on how to be more vigilant, while 54% of those receiving the standard had received such training. However, it should be remembered that they had received this information 5 months prior to this campaign. Secondly, the difficulty of recognizing the standard email as a phish was increased. In this set of emails, the baseline element for recognizability was the fact that the same false sender (risorse.dipendenti@censura.com) was sending the email but the presentation and grammar within the message were improved compared to the first campaign. Lastly, the customization context was changed from the training context to an urgent request to click to confirm your email to receive a Christmas bonus.

In the second campaign, the custom email was the first sent. The reaction to this email led to the campaign being stopped. Some employees asked the Labour Unions for an explanation. The Union requested that management block the dispatch of the emails to avoid confusion as no such bonus was available. The unions did not agree that this was a typical tactic of phishers and the benefits of the simulation would outweigh the confusion and disappointment that the bonus was not real.

As a result of union intervention, the standard mail was not sent in this campaign. Table 2 provides the data on the emails sent in the second campaign and a summary of the response to those emails.

**Table 2.** Details of messages sent and responses in the second campaign.

Second campaign	Custom email	
Total emails sent (1600 went to junk mail)	2700	100%
Received not opened	452	42% of emails in inbox 17% of emails sent
Received and opened	648	24% of emails sent 59% of emails in inbox
Received, opened, link clicked	564	21% of emails sent 51% of emails in inbox 87% of emails opened

### Third campaign

After the problems encountered during the second campaign, for the third campaign, a decision was made to send only one kind of email to all the users. A middle ground was chosen between the standard and a custom phishing mail. An implicit link to the COVID-19 emergency was included and the email offered a gift of an upgrade to Dropbox. The sender was modified to a generic services@censure.com and the content of the mail was presented as an image and not text. Table 3 provides a summary of the messages sent and the responses to these in the third campaign.

**Table 3.** Details of messages sent and responses in the third campaign.

Total emails sent	5198	100%
Received not opened	2900	56%
Received and opened	2298	44%
Received, opened and link clicked	152	3% of emails sent 7% of the opened emails

## Results

Table 4 summarizes the responses to the phishing emails across the three campaigns.

**Table 4.** Summary of responses across the three campaigns.

	Unopened emails		Opened emails		% of opened emails clicked		% of total emails clicked	
Campaign	Standard	Custom	Standard	Custom	Standard	Custom	Standard	Custom
First	64%	38%	36%	62%	18%	88%	7%	55%
Second	–	42%	–	59%	–	87%	–	21%
Third	56%	–	44%	–	7%	–	3%	–

95% confidence intervals (CIs) for binomial proportions were computed with the Wilson method. A two-proportion *z*-test was employed to test for differences between click rates. Click rates are calculated as the percentage of total emails where the link was clicked. Firstly, a significant difference was found in the first round between the click rate of standard (7%) and customized (55%) phishing links (*z* = 9.66, *p* < 0.05). While this comparison could not be made in subsequent rounds for logistical reasons, a significant difference was found between the two rounds of custom phishing emails (*z* = 33, *p* < 0.05) with significantly less being clicked on in the second round (21%) than the first (55%). The same was true for the standard email (*z* = 9.44, *p* < 0.05) with significantly fewer clicks in the third round (3%) than in the first round (7%). In summary, custom emails were more likely to be opened and had a significantly higher click rate than standard emails in the first round. A significant reduction in click rate was found across campaigns for both standard and custom emails but custom emails were still significantly higher. It should be noted that in the second round, only 41% of the sent emails arrived directly into the Inbox, with an extra warning to the target that this email was potentially problematic.

An interesting observation is that the rate of opening each type of phishing email did not change substantially over the campaigns. Obviously, there are many reasons for not opening an email and only some of these staff would know or suspect that it was a phishing attempt. The percentage of unopened emails remains higher for the standard email than the custom email across the campaigns. This demonstrates that customizing the mail to the specific context increases its effectiveness as a phish, that is, more people were curious enough to open the email in the first place. This lack of change in the rate of the opening is curious, as more emails were being sent to the junk mail, meaning that this was not sufficient to reduce the overall rate of opening the emails.

The second interesting observation relates to the percentage of opened emails in which the link was then clicked. In the case of standard mails, this decreased from 18% to 7% between the first to the third campaign. Around half of the staff in the third campaign would have received information about detecting phishing emails after the first or second campaign. In the custom group, the percentage of clicked emails compared to the opened mails stayed approximately the same between the first and second sets of custom mails and substantially less staff would have received follow training after the first campaign. Given that a significant portion of those receiving the phishing email in both the second and third campaigns would have to actively look for this message in their junk mail, which should have alerted more staff to the fact that this email was potentially risky. This could imply that while the mean level of awareness in the case of standard (common or advertising) phishing emails rises over time or was influenced by information received during the phishing exercises, the same is not true for customized phishing emails prepared by the internal ICT who better understand the context of the environment in which they operate and thus can create more persuasive phishing emails.

Statistical analysis of the gathered data in the three cycles of the exercise is not sufficiently strong to allow a conclusion on raising of awareness of the hospital staff to the danger of phishing attacks. However, the calculation of the confidence interval for the difference between the two proportions facilitates consideration of the two groups targeted with the custom and standard mails. The amplitude of the CI, that allows identification of a range of values inside which it is possible to find a real statistical value with a probability imposed a priori (in this case of 95%), was calculated for the custom mail $\left(31\text{\%}<p_{1}-p_{2}<35.4\text{\%}\right)$ an interval wider than that of the CI for the standard mail $\left(2.6\text{\%}<p_{1}-p_{2}<4.8\text{\%}\right)$ . This suggests that the estimate performed in the second case is more accurate than that performed for the first case. Unfortunately, it is not possible to make further deductions on the phishing exercise performed because many factors influenced the experience: firstly, the non-homogeneity of the two selected groups during the whole exercise, and secondly the experiment was conducted in a natural and complex setting where cybersecurity is not prioritized by some, events, such as an improved spam filter came into play, and the organization's reaction to the exercise interfered with the planned campaigns.

## Discussion and lessons learnt

Similar to full-scale live exercises for emergency training, the act of performing an exercise means that real training can take place^29,30^ while simultaneously assessing the current risks within the organization. This is the case with a phishing simulation exercise. However, this was the first time the hospital implemented a phishing simulation and some of the complexities and consequences of running this exercise were unforeseen. The situation was further complicated by the COVID-19 pandemic and the impact this had on the results is not known. However, what is clear is that COVID-19 meant added to the fatigue and workload of all staff. Staff were changing wards and functions, new wards and clinics were opening. Staff were required to learn about COVID and many websites emerged leaving staff facing an “infodemic.”^31^ In addition, more phishing emails were aimed at healthcare organisations.^32^

Undertaking a phishing simulation is a major task and must be undertaken with the full cooperation of the IT department, and in this case with the support of a consultancy company. The simulation was not a research exercise, rather it was a real risk assessment undertaken by the hospital. Many lessons were learnt during this attempt to run a simulated phishing campaign. The results suggest that a general phishing email is easier to detect and ignore than a customized message but the lessons to learn from this case study are more far reaching. This discussion is shaped around the lessons learnt.

### Lesson 1: There are many hidden costs and complexity to consider which requires a full risk assessment

There are many hidden costs and complexities associated with running a phishing simulation within a large organization such as a hospital, which must be well managed if a return on the investment is to be achieved. These include recognizing which staff functions need to be aware of plans and help design the simulation and balancing staff involvement in planning against the effectiveness of the simulation, managing the load on the helpdesk, and lastly managing the impact of the exercise on staff morale and trust.

The risk assessment should consider the well-being of the staff who will experience the content of the messages (e.g. false claims about bonuses) and Human Resources or their union representatives may need to be consulted. Healthcare organizations must ensure that simulated phishing campaigns do not break national employment laws or local agreements with labour organizations.

### Lesson 2: Prepare the helpdesk to support an influx of calls about the exercise

The importance of the Help Desk should not be underestimated. The Help Desk needs to prepare for an influx of alerts during the campaign. It would be easy for the Help Desk to be overwhelmed, which could undermine the effectiveness of the exercise. It may be necessary to employ more staff over the campaign period. Naturally, this creates an administrative workload and has financial implications of hiring temporary staff.

### Lesson 3: Ensure that the right staff are included in the planning and risk assessment

While previous research has focused on the need to engage board members in cybersecurity^33^ decisions, this study highlighted that a broader involvement of staff representatives is required to enable effective phishing simulation. The intervention by the staff Unions, during the second phase, teaches us that an activity of this kind requires the involvement of different hospital functions, including human resources and the unions, during the planning of these events and moreover each function must support these activities: phishing simulation exercises are impossible without the buy-in of hospital management and the complete commitment of all interested parties.

Before introducing phishing simulation into a hospital it would be useful to create a matrix of the services that must be involved in giving consent for specific simulations, following a principle of strictly essential involvement. As our case study shows it is not sufficient to consider only the services that are necessary for the performance of the simulation in itself, but also the General Director, the Chief of ICT, the Help Desk, HR and the Data Protection Officer. To ensure a well-managed campaign, people have important roles to play (see Table 5).

**Table 5.** Roles and responsibilities to run a successful phishing simulation.

Role	People to involve
Ordering simulation and undertaking risk assessment	General Director and Board
Implementation of technical aspects, ensure no security compromises	ICT Management or Chief Information Security Officer (CISO)
Ensure that staff have received appropriate awareness training about the need for phishing simulation, what is surveilled during a simulation, and how to detect a phish.	Training
Management of feedback from/to the users (start, issues arising during, final results and actions arising)	HelpDesk
Staff concerns and contract issues: Ensure that there is no breach of contract/laws or regulations and that the employment of staff is not put at risk.	Human Resources Legal Team
Privacy: Ensure that individuals are not identifiable to the organization and that data of individuals is protected by the external company running the simulation. This will ensure no repercussions on individual staff.	Data Protection Office
Review the content of persuasive messages (rewards or sanctions), and what can be surveilled as part of the exercise (to maintain employee trust)	staff unions, and all relevant departments

### Lesson 4: Find a balance between transparency and effectiveness and educate your staff about the need for phishing simulation

Phishing simulations must balance the various concerns and need for involvement against the potential effectiveness of the campaign. While it is necessary to have the buy-in of different organizational functions, it is also important that as few people as possible know that an anti-phishing campaign is going to take place if a natural response is to be observed. Top management administrative personnel are the most targeted by ‘whale phishing’ which is on the increase,^34^ most likely to fall for a phish^35^ and therefore will gain the most benefit from being subjected to the simulation. Thus, is it important that the simulation target higher levels of hospital administration and a careful balance are required between revealing sufficient information to obtain consent for the simulation while withholding details that would reduce the effectiveness of the simulation.

Transparency is important. When people are unaware of simulated phishing campaigns, they feel like they are under surveillance. That makes the security team – the very team you want your staff to turn to for help – the enemy. Workplaces are all about trust. And trust is a fragile thing. There is a need to be clear, open and transparent about the purpose of your approach and what it means for your staff if a backlash is to be prevented. Awareness training is necessary to ensure that staff understand *why* phishing simulations are necessary and what they will and won’t monitor.

### Lesson 5: Customisation is effective but must be realistic and acceptable

Our results suggest there is a substantial difference in response rates to standard phishing emails, and those customized to the specific hospital context. Therefore, a crucial consideration is how targeted a message should be and how much inside knowledge of a company's socio-technical system should be applied. Such personalization of phishing messages is not simple and may meet resistance not only from the labour unions and staff, as happened in the present use-case, but also by some of the companies that offer phishing exercises as a service. The plan devised for this simulation took into account that an increasingly sophisticated attack is possible and that simulations should take local knowledge into account to ensure that lessons can be learned from the phishing simulation. As can be seen from our results, the majority of staff can identify generic phishing emails. The same was not true of targeted phishing emails where over 50% of staff clicked on the links in the first campaign, which only reduced to 21% when detection was aided by a spam filter. This kind of targeted approach is not typical of the companies that offer phishing simulation services, even though they are technically able to perform these.

### Lesson 6: Customization may work better targeted at job roles or departments

In addition to customizing to a particular setting, in this case, a hospital training schedule and time of year, the variability of staff within the hospital must also be considered. With 6000 staff, there may be a need to differentiate between staff, rather than launch the same campaign on all staff. For example, the fact that some hospital staff had completed online training may make these people less prone to open emails that indicate they have not performed this task. In contrast, some campaigns may work for all the hospital staff, for example, most people are sensitive to bonuses, especially in special periods such as Christmas. However, more work is needed to understand the relationship between the target and the message content and what is most effective.

An alternative approach to mass phishing emails to the whole organization maybe more realistic, and some small quantities of specific phishing mails triggering on particularly sensitive aspects for some crucial departments such as Purchasing or the Internal Pharmacy. This kind of approach could make the difference at the moment when the organization was subject to ‘spear’ or worse still ‘whale’ phishing attacks, which are targeted by definition.

### Lesson 7: Understand what makes a phishing email difficult to detect and easy to fall for

We have moved beyond phishing emails being easily identifiable by poor grammar and misdirected content. Many different persuasion techniques are used in phishing attacks,^36^ evaluations are starting to show how some approaches are more successful than others.^37^ We must now understand more about which persuasion techniques are being adopted and which ones are most effective within different contexts. Phishing simulations need to consider the appropriateness of different persuasive techniques and messages for different staff. Healthcare organizations should be considerate that such deception and/or persuasive techniques may affect industrial relations and trust in the organization.

Future work would also benefit from a more systematic approach to defining the difficulty of detection, perhaps through utilizing a phishing scale.^38^

### Lesson 8: Educate your staff about what to look for and how to do it (beyond poor grammar)

The phishing exercise was considered to be both a risk assessment and a training exercise, there was no expectation of reaching perfection, and the aim was not to make it impossible to recognize a phish but to push the level of difficulty to better mimic the type of targeted phishing attacks that could be conceived. The phishing emails had to contain some elements of recognizability, to allow the user to develop awareness of such elements. Two elements of recognizability, some grammatical errors and a certain ‘fuzziness’ of the images, were present. But the principal message sent to all the users was to be wary of all mail coming from external domains but with a name similar to that of the hospital. Naturally, this presumes that all staff know the constituents of an email address and the correct email for any person from which they receive an email. Knowledge is necessary but not sufficient to drive behaviour, which is why awareness training needs to be complemented with the simulation exercises to assess behaviour rather than knowledge.

Since the domain of the Hospital is a first-level national domain (‘.it’), the phishing e-mails were sent from different domains like for example ‘.com’. Knowing this, one of the best investments a hospital can make is to buy domains similar to the official domain. After this experience the hospital bought similar domains, to prevent such attacks in the future.

The domain in phishing is important to the success of a phishing email, thus when the domain is out of view as in emails received on smartphones, such as the iPhone or from Outlook on Android (Android has corrected this problem in February 2020), their success is more often guaranteed. In these phishing attempts the complete address is visible only after clicking on the address, but how many users do that? This study did not look at the platforms on which staff received the emails. Future work should ensure that such data is captured so that the influence of different platforms and contexts of use can also be assessed.

### Lesson 9: Explore whether other factors affecting phishing susceptibility can be reduced

There are many factors that affect an individual’s likelihood of falling for a phish. These include factors such as workload and fatigue, lifestyle and routine, impulsivity and trust.^39–41^ From our data it is impossible to understand which of these mechanisms is the cause of the falling for a phishing scheme, but an interesting observation comes from the discrepancy between the numbers of the ‘clickers’ of the standard emails and the custom emails. The difference between these two types of emails lies in the persuasiveness of the object of the mail: in one case a hypothetic chastise and in another a company bonus.^36,37^ More work is required to understand the relative persuasiveness of different targeted messages as well as the relative difficulty in detection.^42^

Whatever the reasons, however, it is evident that hospital staff are already fatigued and overloaded and we cannot constantly run phishing simulations. While staff should be aware of the basic rules to recognize generic false emails (spelling errors, poor layouts, unfocused images, also in relation to the institution of the sender), the real challenge is to eliminate ‘automatic habits’. One must influence the users to ask themselves some questions relative to the identity of the sender and to call the Help Desk for help, etc. Basically, to consider the ‘virtual’ space where they are working at the same level of the ‘real’ world they are living in, overcoming the well-known credibility bias that is present towards technology systems.^43^

Anti-spam filters, the tagging of mails coming from outside the Hospital and the training of the staff may and should help in recognizing phishing emails, but ultimately the choice of whether to click or not lies with the receiver and we need to be more considerate of their workload and pressures that lead to clicking.

### Lesson 10: Enact the phishing campaign as quickly as possible

While planning may take some time, the actual campaign must be enacted quickly. In this case, the simulation took place over two to three days, which may be too long. Too much time will allow news of the exercise to spread by ‘word of mouth’ – telling colleagues what to look out for. Not only is it important that the news of the upcoming simulation not be propagated, nullifying the effect of the exercise, but also that there is no time to propagate fake news, as happened with the ‘bonus’ email, that created a ‘flurry of misunderstanding’ in the relative management offices. ‘Word of mouth’ is also a valid defence tool, ensuring that other members of staff do not make the same mistake, and while it can be an effective defence mechanism, it can interfere with phishing simulations.

### Lesson 11: Ensure supporting technology is trusted by staff

This case study highlighted, albeit by coincidence rather than planning, that the risk of phishing can be partially mitigated by the technological component utilized to defend a company. In this case, anti-spam filters redirected 30% of the phishing emails to junk mail. This happened only because the anti-spam filters received an elevated number of emails from the same address in a very short time but cannot, therefore, be completely relied on. Moreover, before their automatic activation, they allowed the passage of 20% of spam emails. Large volumes of email are the most basic phishing approach and it would be tempting to configure the infrastructure to let all simulated phishing messages reach the staff, but this would have security implications for the hospital and leave them vulnerable during such exercises. A smaller number of emails, well addressed, would have passed through undisturbed. In this context, the most recent technological instruments still have difficulty in blocking spear or whale phishing and this is potentially where the organization is most vulnerable. Alongside this, training to recognize phishing must be kept up to date and simulated attacks must be contextualized.

Spam filters need to be optimized so that users can trust that what is there is dangerous. The more that staff are required to move legitimate emails from junk folders, the more likely they are to wrongly move phishing emails. However, as a risk assessment, the security of the organization should not be altered for the exercise. Security is a holistic problem where technological and human defences must work effectively together.

### Lesson 12: Management must be committed to the exercise – this **is** not a game

A phishing attack can put a hospital's infrastructure at risk, which in turn risks patient privacy and/or lives. Currently, technology cannot reliably detect phishing attacks and so human behaviour must be relied on. Phishing simulations assess the risk position of an organization posed by staff. Management must see this as a serious issue and commit to the phishing simulation, as they would any other emergency training simulation – it is not simply a game. Effective simulations exploit human vulnerabilities, and if the simulation does not do the same it will not serve as effective training. In this case, the management stopped the phishing simulation exercise when the Unions got involved rather than convincing them of the importance of the exercise.

Not all hospitals are willing to do this, and few research exercises take place ‘in the wild’ which limits their effectiveness. Other attempts to investigate phishing susceptibility within hospitals have used alternative means of measurement such as a phishing questionnaire^2^ Whilst research projects offer more control over the demographics of participants and control over experimental conditions which facilitates statistical comparisons of participants by demographic and/or experimental conditions, they are not a reliable measure of behaviour ‘in the wild’.

### Lesson 13: Communicate the results back to staff and management

To ensure transparency to staff, the results of the exercise should be summarised back to staff, and ensure that they are aware of the current position, what the results are used for and the fact that even one person falling for a phish leaves the hospital vulnerable to attack. Such communication helps to ensure transparency (lesson 4).

Management also needs to receive the results so that they can update their risk assessments, and decide on an improvement plan, should one be needed.

### Limitations

Limitations of the performed exercise are evident and easily recognizable ‘a posteriori’. This simulation ran across the start of the COVID-19 pandemic and it is not possible to define the extent that this affected acceptance of the approach or the results. COVID-19 limited the opportunity for feedback to staff and management as it was felt that they were already overloaded with the pandemic.

It is the case that personnel are numerous and diverse, with a different understanding of phishing and/or vulnerability to phishing. To ensure that staff were not ‘blamed’ for falling for a phish, identities were not tracked, however, this limits the amount of analysis that can be carried out to see if behaviour changes in individuals over the campaigns or if there is a particular demographic that is vulnerable to these attacks. The study also did not take into account any new personnel hired after the start of the first campaign.

The main limitation was being unable to complete the three campaigns, with each comparing a standard and generic phishing email. This resulted from the adverse reaction to the enticement of a Christmas bonus, that was not real. In hindsight, a full risk assessment should have been carried out to identify potential unintended consequences of phishing campaigns in general and the specific enticements used. Management should then have been informed before the campaign was started.

As part of the risk assessment, a pilot of the campaigns would have been beneficial. However, in an attempt to ensure that staff were not aware of the specific campaigns, the campaigns were not piloted before their release to the whole hospital. Perhaps a better compromise would have been to involve a small number of individuals to pilot the phishing emails and gauge their reaction to them.

The different employee groups and unions representing them were not consulted before the campaigns. To avoid the threat of industrial action, a general consultation is required with the unions to discuss the costs and benefits of phishing simulations in general, and have unions and employees onside before commencing campaigns. It may be necessary to raise the awareness of staff and unions about the need for this type of phishing training.

## Conclusions

Hospitals and other healthcare organizations face a variety of challenges to their cybersecurity one of which is phishing attacks. Hospitals must remain vigilant as these are not easy to manage – even if staff want to identify phishing emails, it does not mean they can. Training employees to recognize and counter phishing is important. One way to do this can be through phishing simulations but this is not without its problems. Not all phishing simulations are equal, some are more difficult to detect than others. Customization of phishing emails to the specific work context provides stimuli that are relevant to the current attack landscape, however, they carry with them ethical concerns. Simulation training adds to the workload of an already fatigued workforce and ironically, the workload is positively associated with clicking on phishing emails. Alongside this, contextualization, and its associated false threats or promises, can create anxiety and such deception can be ethically questionable. We must also be mindful of the different platforms where email is accessed, and the growing use of mobile phones which obfuscate important information in the form of the sender’s email address and the ability to hover over a link to see the true destination.

For phishing simulations to be effective and avoid hidden costs, organizations should carefully consider cross-organization involvement in planning, frequency of repetition, tailoring and targeting of messages, short transmission times while being mindful of the stress and workload they are creating.

New systems which perform phishing exercises from within the hospital structures may better fulfil the needs and better enable the tailoring of the phishing emails that are sent. This in turn will help facilitate the identification of the reasons why staff continue to fall for phishing emails. Continuous online training can help to some extent but given the levels of workload and fatigue, it is a problematic solution that adds to the workload of an already fatigued workforce, which has been identified as a factor positively associated with clicking on phishing emails.^6^ Users will benefit from improved technical support in terms of more trustworthy removal of phishing from their inbox (reducing their workload), and ways of alerting the user to suspicious emails which highlight what to check on the email and take advantage of the ‘nudging’ toward better cybersecurity behaviour^11^ and other suggested approaches to having a more proactive approach to cybersecurity in healthcare.^44^

Our data clearly show that many users have developed, probably due to their daily experience, and ability to recognize conventional mass phishing. However, the same is not true for a more serious and targeted phishing message. This reported case study might be helpful for other Hospitals to learn from and improve their approach to phishing simulation to guarantee better cybersecurity management.
